# Treating Intracranial Abscesses in Rats with Stereotactic Injection of Biodegradable Vancomycin-Embedded Microparticles

**DOI:** 10.3390/pharmaceutics12020091

**Published:** 2020-01-22

**Authors:** Yuan-Yun Tseng, Ching-Wei Kao, Kuo-Sheng Liu, Ya-Ling Tang, Yen-Wei Liu, Shih-Jung Liu

**Affiliations:** 1 Division of Neurosurgery, Department of Surgery, Shuang Ho Hospital, Taipei Medical University, Taipei 11031, Taiwan; britsey@gmail.com; 2 Department of Surgery, School of Medicine, College of Medicine, Taipei Medical University, Taipei 11031, Taiwan; 3 Department of Anesthesiology, Chiayi Chang Gung Memorial Hospital, Chiayi 61363, Taiwan; seth7200@gmail.com; 4 Department of Mechanical Engineering, Chang Gung University, Tao-Yuan 33302, Taiwan; vellick27candy@gmail.com; 5 Department of Thoracic and Cardiovascular Surgery, Linkou Chang Gung Memorial. Hospital, Taoyuan 33302, Taiwan; liuks@me.com (K.-S.L.); anglevvings@gmail.com (Y.-W.L.); 6 Department of Orthopedic Surgery, Chang Gung Memorial Hospital-Linkuo, Tao-Yuan 33305, Taiwan

**Keywords:** brain abscess, stereotactic, poly(lactide-*co*-glycolide acid), electrospraying, drug delivery, vancomycin

## Abstract

Brain abscesses are emergent and life-threating despite advances in modern neurosurgical techniques and antibiotics. The present study explores the efficacy of vancomycin embedded to 50:50 poly(lactic-*co*-glycolide acid) (PLGA) microparticles in the treatment of brain abscess. The vancomycin embedded microparticles (VMPs) were stereotactically introduced into the cerebral parenchyma in *Staphylococcus aureus* bacteria- induced brain abscess-bearing rats. Experimental rats were divided into three groups: group A (n = 13; no treatment), group B (n = 14; daily vancomycin injection (5 mg intraperitoneally), and group C (n = 12; stereotactic introduction of VMPs into the abscess cavity). Group C exhibited no inflammatory response and significantly increased survival and reduced mean abscess volumes (*p* <0.001) at the eighth week, compared with other groups. Vancomycin delivery via a biodegradable PLGA vehicle can easily attain Area Under the Curve (AUC)/minimum inhibitory concentration (MIC) ratios of ≥400, and strengthens the therapeutic efficacy of antibiotics without provoking any potential toxicity. Biodegradable VMPs are a safe and sustainable drug delivery vehicle for the treatment of brain abscess.

## 1. Introduction

Brain abscess, a dynamic focal form of intracranial suppuration, is an emergent and life-threating illness [1,2]. The incidence of brain abscess is approximately 1–2% in developed countries, whereas in developing countries it is much higher, at approximately 8–10% [3,4,5]. Brain abscesses can generate primarily from an adjoining structure infection (e.g., otitis media, oral cavity, dental caries, mastoiditis, or sinusitis), or can be secondary to hematogenous dispersal from a distant site, after skull trauma or neurosurgical procedures, and, rarely, after meningitis. In at least 15% of cases, abscesses occur with unknown mechanisms [2,6]. Despite the development of newer antibiotics and advanced neurosurgical techniques, including stereotactic brain biopsy and aspiration, improved culturing techniques for recognizing infectious organisms, and progressive neuroimaging procedures, the overall outcome of brain abscess remains a challenge for the neurosurgical community with substantial patient fatality rates [1,7,8].

Successful treatment of brain abscesses often requires the combination of adequate antibiotics and surgical drainage for both diagnostic and therapeutic purposes [2,7]. Nonsurgical treatment with antibiotics alone could be applied when the abscess is in the cerebritis stage or less than 3 cm in diameter without capsule formation [9,10,11].

Pathogenic organisms differ on the basis of the site of origin. In the preantibiotic era, *Staphylococcus aureus* was the most common organism isolated from brain abscesses [4,5]. With the widespread use of penicillin and other advanced antibiotics, the incidence of *Streptococcus* spp. has increased and replaced *Staphylococcus* spp. as the most common organisms in some studies [5,12]. Systemic drug treatment of central nervous system (CNS) diseases, such as brain abscesses, tumors, and neurodegenerative diseases, is a daunting challenge because of the distinctive protective system, mainly the blood–brain barrier (BBB) and the blood–cerebrospinal fluid barrier [13]. The distinct protective structure of the CNS also confines the delivery of therapeutic agents into the brain parenchyma. Many categories of systemic therapeutic agents, including antibiotics and chemotherapies, fail to reach effective levels in the CNS even while the systemic concentrations achieve toxic concentrations and severe side effects [14,15]. The instructed therapy period of parenteral antibiotic treatment for brain abscesses is prolonged, basically 4–8 weeks according to the treatment result and followed by neuroimaging [3,4,7]. The prolonged therapeutic period undoubtedly exacerbates antibiotic toxicity and/or resistance. Local delivery of antimicrobial agents to treat brain abscesses provides the advantage of transporting high concentration drugs to the target site with minimum systemic side effect. Nevertheless, to the best knowledge of the authors, no research has investigated the use of antibiotic-loaded microparticles for the treatment of brain abscesses.

In the present study, we embedded vancomycin to 50:50 poly(lactic-*co*-glycolide acid) (PLGA) microparticles by adopting the electrospraying technique. The vancomycin-embedded microparticles (VMPs) were stereotactically introduced using a Neuros syringe into the brain parenchyma of healthy rats following a simple burr hole operation. The in vivo release profiles of vancomycin were explored. In addition, *S. aureus* bacteria were stereotactically injected into the brain parenchyma to create brain abscesses in the rats. After brain abscess formation was confirmed using magnetic resonance imaging (MRI), the brain abscess-bearing rats received pus aspiration and were randomly divided into three groups. The rats in group A received no other treatment after pus aspiration, whereas the rats in group B received daily vancomycin injections (5 mg) intraperitoneally. The rats in group C received VMPs (2.5 mg PLGA microparticles containing 0.5 mg of vancomycin) stereotactically introduced into the abscess cavity through the same burr hole after pus aspiration. The survival rate in each group was evaluated, and the abscess volumes were estimated using regular MRI examinations. Tissue reactions were also histologically examined.

## 2. Material and Methods

### 2.1. Fabrication of VMPs

The poly(lactide-*co*-glycolide acid) polymer Resomer RG503 (lactide:glycolide, 50:50) (molecular weight: 33,000 Da) was purchased from Boehringer Ingelheim (Ingelheim am Rhein, Germany). Vancomycin was commercially acquired from Sigma-Aldrich (Steinheim, Germany).

Microparticles incorporated with vancomycin were prepared using a laboratory-made electrospraying device featuring a syringe and nozzle (internal diameter = 0.60 mm), ground electrode, collector, and high-voltage supply. PLGA and pharmaceuticals (600 mg of PLGA, 50 mg of vancomycin) were first mixed with 1 mL of dichloromethane by a magnetic stirrer at ambient temperature with a speed of 900 rpm for 1 h. During the electrospraying process, the solution was transported utilizing a pump with a flow speed of 0.9 mL/h. The nozzle was connected to a high-voltage supply that has a positive DC voltage of 8 kV. The travel distance from the nozzle tip to the ground electrode was 13 cm. All electrospraying experiments were completed at a temperature of 25 °C and a relative humidity of 60%.

### 2.2. Fourier-Transform Infrared Spectroscopy

Fourier-transform infrared (FTIR) spectroscopy was employed to assess the spectra of pure PLGA microparticles and vancomycin-embedded PLGA microparticles. The FTIR assay was completed using a Nicolet iS5 spectrometer (Thermo Fisher Scientific, Waltham, MA, USA) that has a 4 cm^−1^ (32 scans) resolution. Electrosprayed particles were compressed into potassium bromide (KBr) discs, and the spectra were monitored within the 400–4000 cm^−1^ range.

### 2.3. Characterization of Electrosprayed Particles

The morphologies of the electrosprayed microparticles were observed under a JSM-7500F field-emission scanning electron microscope (JEOL, Tokyo, Japan). The zeta-potential and size distribution of the particles were measured by an ELSZ-2000 Zeta potential and particle size analyzer (Otsuka Electronics, Osaka, Japan).

### 2.4. In Vitro Release

The release profiles of vancomycin from electrosprayed particles were evaluated by the in vitro elution method. VMPs were placed in glass test tubes (one sample per test tube, *n* = 3) with 1 mL of phosphate buffer solution (PBS) (0.15 mol/L, pH 7.4). The glass test tubes were incubated at 37 °C for 24 h before the VMPs eluent was gathered and assayed. New PBS was then added for the next 24-h period, and this procedure was duplicated for 30 days. The drug concentrations in the eluents were assessed using a Hitachi L-2200 high-performance liquid chromatography analysis (Tokyo, Japan). A Symmetry C8, 3.9 cm × 150 mm HPLC column was utilized for the assay. The mobile phase used for vancomycin contained 0.01 mol heptanesulphonic acid (Fisher Scientific U.K. Ltd., Loughborough, UK) and acetonitrile (Mallinckrodt, St. Louis, MO, U.S.A.) (85/15, *v*/*v*). The absorbency was monitored at a wavelength of 280 nm and the flow rate was 1.4 mL/min.

### 2.5. Surgical Procedure

All experimental procedures gained approval from the Chang Gung University Institutional Animal Care and Use Committee (CGU107-028, approval date: 29 October 2018), and animal care adhered to the guidelines of the Department of Health and Welfare, Taiwan. A total of 75 male Sprague Dawley rats (with a weight ranging from 200 to 250 g) were commercially acquired from BioLASCO Taiwan Co., Ltd. (Taipei, Taiwan). The experimental rats were first quarantined at the animal center for seven days prior to undergoing procedures.

Thirty rats were employed in the in vivo drug release study. Each rat received inhalation anesthesia with halothane. The anesthetic depth was evaluated using either corneal reflex tests or tail pinch. After the postorbital region was shaved and sterilized, a small longitudinal scalp incision of approximately 10 mm was performed between the ear and the eye. The scalp fascia and muscle were analyzed with a scalpel. A burr hole (about 1.5 mm in diameter) was drilled just above the temporalis muscle using an electric burr. The rats were positioned in a stereotactic instrument (Model: 68001, RWD Life Science Inc., San Diego, CA, USA) after meticulous hemostasis. Then, 2.5 mg of biodegradable PLGA microparticles embedded with 0.5 mg of vancomycin (VMPs) were mixed with 10 µL of dimethyl sulfoxide (DMSO) and were slowly injected into the brain parenchyma (lasting >3 min in total) using a 50 µL Neuros syringe pierced 3 mm into the core of the burr hole. The operative wound was sutured with 3-0 nylon. After the animals recovered from anesthesia, they were sent back to the residential center. Rats that displayed intraoperative cerebral injuries or infections were eliminated from the research.

### 2.6. In Vivo Antibiotic Pharmacokinetics

**Figure 1:**
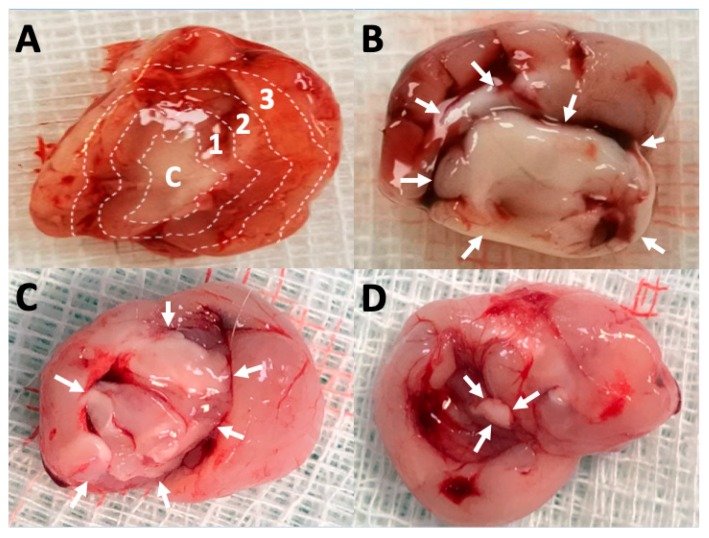
Degradation of VMPs. (**A**) Injected vancomycin-embedded poly(lactide-*co*-glycolide acid) microparticles (VMPs) resulted in a white core, and the brain parenchyma was divided to zones 1–3 (each zone thickness ~2–3 mm) for tissue sampling. C: core, 1: zone 1, 2: zone 2, and 3: zone 3. Overall appearance **(B**) 1 week, (**C**) 4 weeks, and (**D**) 8 weeks after VMP injection into the brain parenchyma. Injected VMPs were denser and greater (indicated by white arrows) during the first few weeks and degraded with time.

The rats were randomly divided into nine groups (3 days and 1, 2, 3, 4, 5, 6, 7, and 8 weeks), each of which comprised three or four rats. Carbon dioxide inhalation was used to euthanize the animals. Approximately 0.5 mL blood sample was collected utilizing 1 mL syringes via a cardiac puncture. Cerebral parenchyma injected with particles was removed surgically. Cerebral parenchyma samples 8 × 8 mm^2^ in area and 8–10 mm in thickness were divided into three zones, namely zones 1–3 from the core (the tract of injected VMPs) outward from the center of the brain, and each zone was about 2–3 mm thick (Figure 1A). 

Cerebral parenchyma samples weighing approximately 50 mg were obtained from each divided zone. All samples (blood and various brain tissue zones) were gathered at 3 days and 1–8 weeks. The tissue samples were collected using sonication for 20 s, followed by centrifugation. In addition, blood was gathered and maintained at −80 °C. Concentrations of vancomycin in gathered samples were determined using the HPLC assay.

### 2.7. Bacterial Preparation

*S. aureus*, the most widespread etiology bacteria identified in traumatic and iatrogenic abscesses [7,12,16], was selected as the pathogen in this study. The microorganisms were cultured in static at 37 °C for 24 h in 12 mL of broth (LB broth, GIBCO, Thermo Fisher Scientific, Inc., Waltham, MA, USA). Prior to the experimental procedures, the cultured media first underwent centrifugation at 3000 g for 15 min, and the supernatant was poured off. This stock solution was diluted 10 fold serially. A standard microbiological plating/counting method was then utilized to decide the number of bacteria present in the solutions. The final levels of viable pathogens were in the range of 1 × 10^6^ to 2 × 10^6^ colony-forming units (CFU)/µL.

### 2.8. Brain Abscess Model Creation and Treatment

Using the aforementioned procedure, the brains of 45 more healthy male Sprague Dawley rats were injected with 10 µL of isotonic saline containing 10^7^ *S. aureus*. Approximately 14 days after bacteria solution injection, the rats received brain T1- and T2-weighted MRI examinations to confirm that the brain abscess models were successfully established (excluding intracranial hemorrhage). The abscess-bearing rats were anesthetized with halothane inhalation and the scalp wounds were reopened. The abscess-bearing rats were secured in a stereotaxic instrument, and a Neuros syringe was again pierced 3 mm into the core of the burr hole, and approximately 10 µL of pus was withdrawn from the abscess of each abscess-bearing rat. The abscess-bearing rats were randomly divided to three groups. A 10-µL DMSO solution was injected into the brain abscess cavity in groups A and B. The rats in group A did not receive any treatment, and the rats in the group B received a 5 mg vancomycin injection intraperitoneally daily until the rats died. The abscess-bearing rats in group C received the injection of VMP/DMSO solution. T2-weighted MRI images were obtained as a standard to recognize abscess volumes at week 1, 2, 4, 6, and 8 after treatment. The abscess volumes were re-established and calculated using the open-source software OsiriX. At post-treatment week 6, at least one animal in every group was euthanized, and the cerebral parenchyma was meticulously extracted for pathological and histological analysis. Specimens were subjected to hematoxylin and eosin staining before using light microscopy.

### 2.9. Statistical Analysis

The data were obtained from the samples and expressed as means ± standard deviations. The overall survival among rats in the three groups were analyzed using the Kaplan–Meier scheme. Abscess volumes were calculated and analyzed utilizing a paired sample t-test and one-way analysis of variance. Significance was determined utilizing paired sample t-tests using Stata SE (version 12.0, StataCorp, College Station, TX, USA); *p* < 0.05 was significant.

## 3. Results

### 3.1. Morphology of the Microparticles

**Figure 2:**
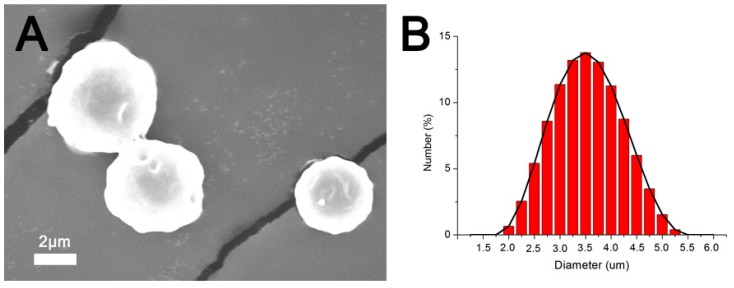
Vancomycin-embedded poly(lactide-*co*-glycolide acid) microparticle (VMP). (**A**) Scanning electron microscopy (SEM) image of VMPs (x 6000) and (**B**) particle size distribution.

Figure 2A presents an SEM image of the VMPs. The particles exhibited relatively spherical geometry. Figure 2B shows the size distribution of the particles. The mean diameter of the VMPs was 3.7 ± 0.91 µm. Meanwhile, the zeta potential of the particles was measured to be −25.82 ± 2.6 mV.

### 3.2. FTIR Spectroscopy

**Figure 3:**
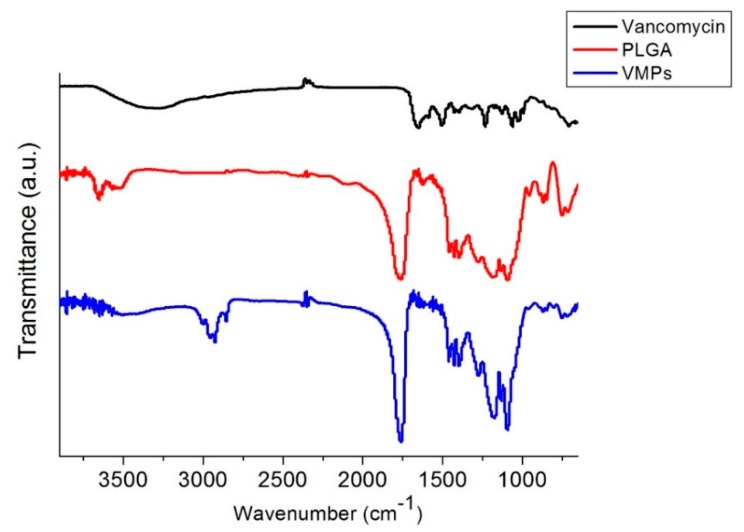
Fourier-transform spectra of the pharmaceuticals, pure poly(lactic-*co*-glycolide acid) (PLGA), and VMPs.

Figure 3 displays the FTIR spectra of vancomycin, virgin PLGA microparticles, and VMPs. The peak formation at 3200–3500 cm^−1^ of VMPs might be attributable to the N–H bonds of vancomycin [17]. The absorption at 1760 cm^−1^ (C=O bond) was enhanced by the embedment of antibiotics. Additionally, the new peak near 1180–1360 cm^−1^ was attributed to the vancomycin C–N bonds. Thus, the FTIR spectra assay indicated that vancomycin was successfully incorporated into the VMPs.

### 3.3. In Vivo Elution Behavior of Vancomycin From VMPs 

The overall appearances of brain parenchyma 1, 4, and 8 weeks after VMP injection are displayed in Figure 1B–D, respectively. Injected VMPs were denser and greater (indicated by white arrows) during the first few weeks and degraded with time. Only a small amount of residual multiple-antibiotic-embedded microparticles was exhibited at the end of study (8 weeks). The scalp and brain tissue were relatively clear, and no infection (exudate, pus, or granulation) was observed.

**Figure 4:**
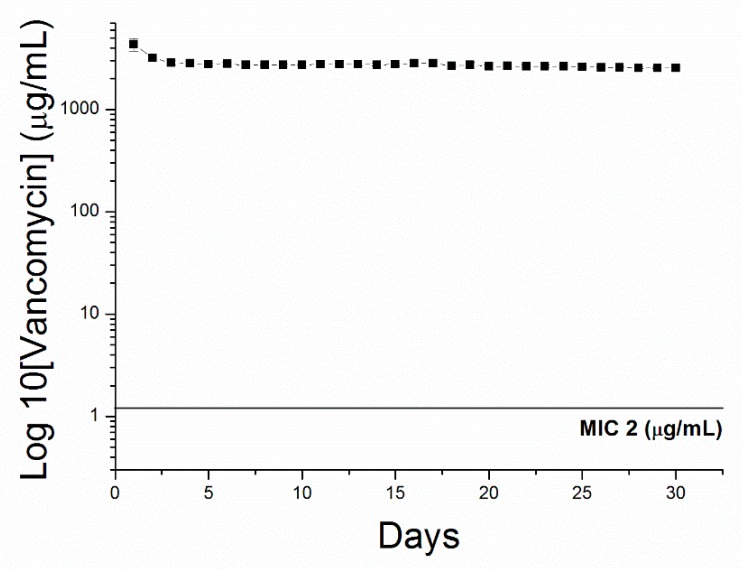
In vitro release. In vitro release of vancomycin from biodegradable antibiotic-embedded microparticles (solid line is the minimum inhibitory concentration (MIC) of vancomycin).

**Figure 5:**
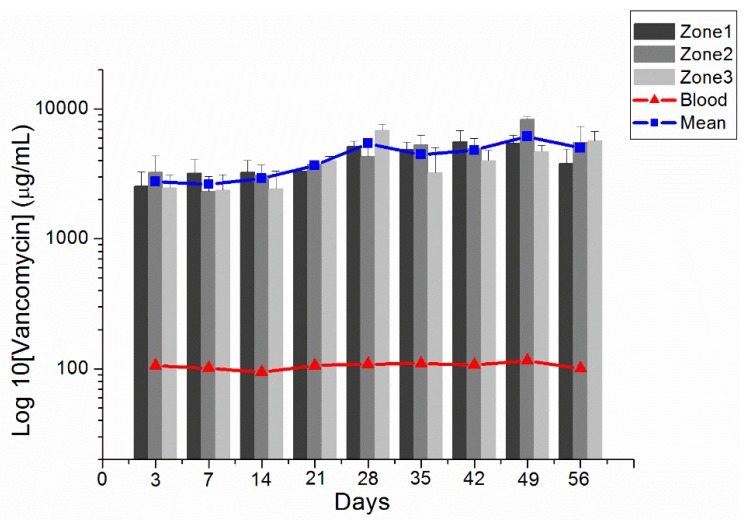
In vivo release. In vivo release of vancomycin from biodegradable antibiotic-embedded microparticles and concentrations in various zones of the brain parenchyma and blood (error bars indicate standard deviation).

Figure 4 shows the in vitro release profiles of vancomycin from the electrosprayed particles. The results suggest that VMPs released high concentrations of antibiotics for more than 30 days. In vivo vancomycin concentrations were analyzed for 8 weeks utilizing an HPLC method. The rats that died during the perioperative period (due to anesthetic overdose or enormous blood loss), those with brain injuries or wounds, and those with brain, peritoneal, or systemic infections were excluded. Subsequently, four rats on postinjection day (PID) 3, three on PID 7 (1 week), three on PID 14 (2 weeks), four on PID 21 (3 weeks), three on PID 28 (4 weeks), three on PID 35 (5 weeks), three on PID 42 (6 weeks), four on PID 49 (7 weeks), and three on PID 56 (8 weeks) were obtained for drug concentration assay. Figure 5 presents the release curves of vancomycin in different zones of the brain tissue and in the blood. Vancomycin concentrations in brain tissue swiftly reached high levels on PID 3 (2639.31 ± 920.75 µg/mL), reached maximum concentration (6821.92 ± 826.16 µg/mL) on PID 42, and were maintained from 2639.31 ± 920.75 µg/mL to 6821.92 ± 826.16 µg/mL for over 8 weeks. Vancomycin levels were much lower in blood (ranging from 91.19 ± 6.82 to 110.47 ± 15.27 µg/mL). Furthermore, the near-core area (zone 1) did not exhibit higher concentrations than the far-core area (zone 3) during the initial few weeks. No differences between the near-core and far-core zones reached significance at any time point.

### 3.4. Survival Rate

**Figure 6:**
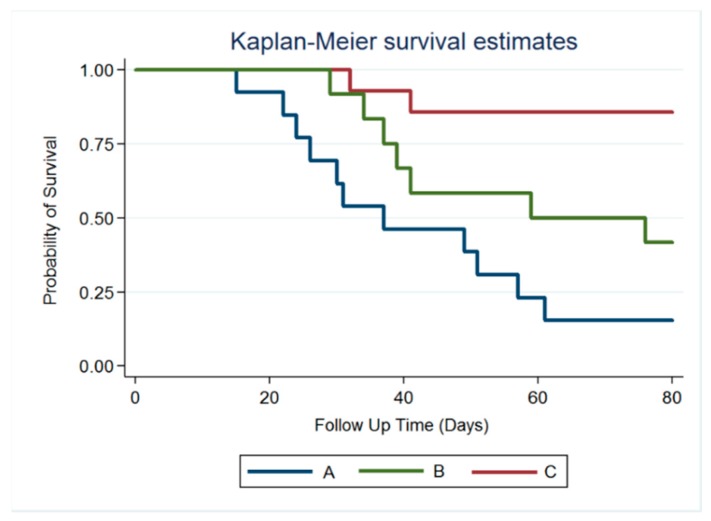
Survival curve. Survival rates for all three groups were analyzed using the Kaplan–Meier method. No difference was noted between groups A and B (*p* = 0.08). The overall survival rate was significantly higher in the vancomycin-embedded poly(lactide-*co*-glycolide acid) microparticle treatment groups (group A vs. group C, *p* < 0.001; group B vs. group C, *p* < 0.001).

Brain abscesses were successfully created and verified using MRI in 39 rats (13, 14, and 12 rats in groups A, B, and C, respectively). In group A, nearly half of the rats (6/13) died 4 weeks after stereotactic abscess aspiration and injection of 10 µL of normal DMSO solution into the brain abscess cavity. At the end of the study (week 8), 11 rats died, and 2 rats with small brain abscesses survived. In group B, the rats received abscess aspiration followed by a daily intraperitoneal injection of vancomycin (5 mg). One rat died of intraperitoneal infection at week 3. Five rats (of the total 12) in group B died at week 6, and five rats survived until week 8. In group C, 10 µL of VMP/DMSO solution was injected using a stereotactic needle after aspiration of 10 µL of pus. Only two rats died by the end of study; 83.33% animals (10/12) were alive, and no obvious residual abscesses were observed in five rats by using MRI. Figure 6 displays the Kaplan–Meier analysis result at representative survival times. In the VMP treatment group (group C), the mortality of brain abscesses was significantly decreased. The statistical differences between groups A and C and between groups B and C were significant (*p* < 0.001). However, the difference between groups A and B was nonsignificant (*p* = 0.095).

### 3.5. MRI and Tumor Volume

**Figure 7:**
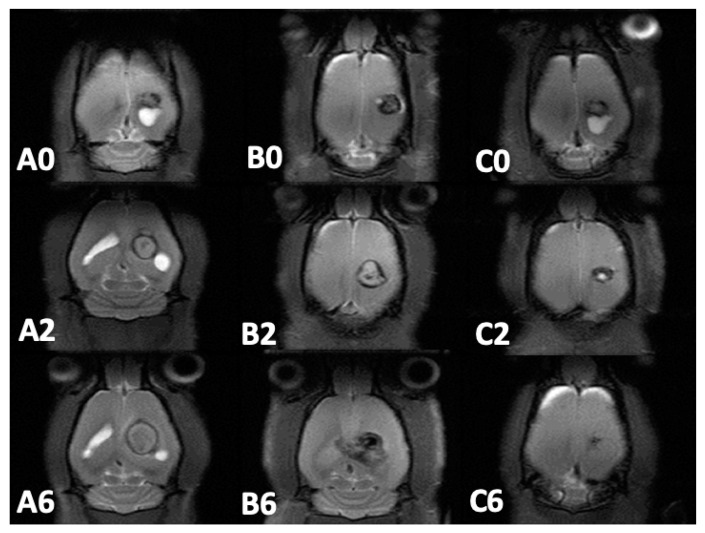
Serial magnetic resonance imaging. The number in the lower left-hand corner of each image indicates the number of weeks after different treatments. No significant differences among pretreatment brain abscesses in groups A (A0), B (B0), and C (C0) were observed. The abscesses in groups A and B enlarged progressively with obvious mass effect. Bilateral ventricle involvement was noted in group A (A2 and A6). The abscess volume increased and grew inwardly (B2) then crossed the midline (B6). The abscess volume decreased progressively in group C after injection of vancomycin-embedded poly(lactide-*co*-glycolide acid) microparticles (C2 and C6), and no perifocal edema was observed. (x 0.8).

Approximately 12–14 days after *S. aureus* injection into rat brains, MRI images (T1- and T2-weighted) were obtained to ensure the successful establishment of brain abscess models. Serial brain MRI examinations were performed prior to the treatment (week 0) and 1, 2, 4, 6, and 8 weeks after treatment in the three groups. The withdrawn pus volumes were 8.6 ± 2.69 µL, 9.0 ± 3.76 µL, and 8.91 ± 2.79 µL in groups A, B, and C, respectively. No significance was observed (*p* = 0.484). Figure 7 exhibits the serial brain MRI of abscess-bearing rats in the three groups. Abscess volumes increased substantially in groups A and B and considerably decreased in group C.

The mean brain abscess volumes before treatment were 51.97 ± 25.72 × 10^−3^ mL, 49.90 ± 20.59 × 10^−3^ mL, and 54.16 ± 21.68 × 10^−3^ mL in groups A, B, and C, respectively. No significance was observed (*p* = 0.287) among the groups. In group A (no treatment), brain abscess volumes increased considerably and reached their maximum (197.45 ± 2143.68 × 10^−3^ mL) at week 4, and nearly half of the rats (6/13) died at week 4. Mean abscess volume decreased to 175.37 ± 163.99 × 10^−3^ mL at week 6. Due to the death of rats with large abscess volumes, the mean abscess volume of the remaining two rats in group A was 19.28 ± 17.83 × 10^−3^ mL. In group B (treatment with intraperitoneal injection of vancomycin), abscess volumes increased substantially. Mean abscess volume reached its maximum (135.16 ± 96.95 × 10^−3^ mL) at week 2 and progressively decreased thereafter. The mean abscess volumes were 86.95 ± 98.43 × 10^−3^ mL and 72.32 ± 86.27 × 10^−3^ mL at weeks 4 and 6, respectively. Nearly half of the rats (5/12) died at week 6. In group C (treatment with VMP injection), the abscess volumes increased indistinctly and reached their maximum (61.03 ± 39.37 × 10^−3^ mL) at week 1. The difference in abscess volumes between the beginning of the study and at week 1 was nonsignificant (*p* = 0.74). Mean abscess volumes decreased progressively and were 47.37 ± 26 × 10^−3^ mL, 38.85 ± 62.59 × 10^−3^ mL, and 15.66 ± 16.87 × 10^−3^ mL at weeks 2, 4, and 6, respectively. The differences in abscess volumes between weeks 2 and 4 and between weeks 4 and 6 were significant (*p* = 0.02 and 0.03 respectively).

**Figure 8:**
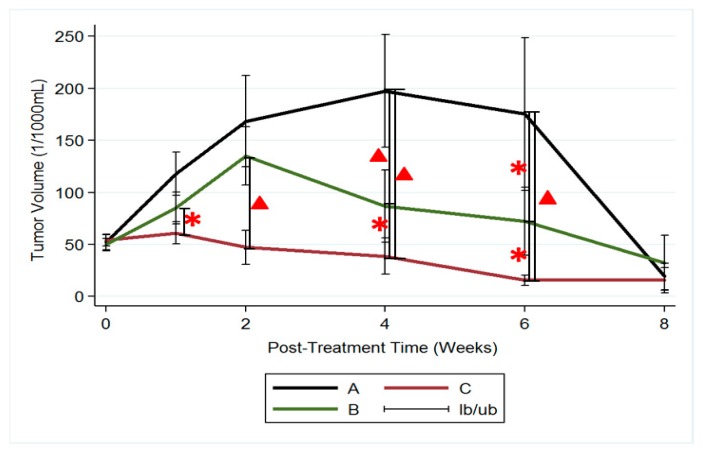
Brain abscess volumes. A repeated-measures mixed model was used to evaluate the brain abscess volume changes in the three groups. Brain abscess volumes in groups A and B increased rapidly and were significantly greater than those in group C, with differences reaching significance (group A vs. group C, *p* < 0.001; group B vs. group C, *p* < 0.001). (▲: *p* < 0.01; *: *p* < 0.05).

Figure 8 presents the results of using the repeated-measures mixed model to assess the mean abscess volume among the three groups. Significant differences were observed between groups A and C and between groups B and C (*p* < 0.001). However, no significant difference was noted between groups A and B (*p* = 0.4).

### 3.6. Pathologic Findings

**Figure 9:**
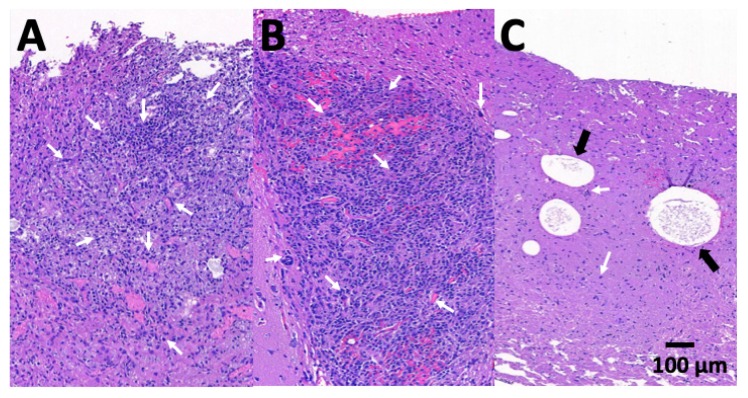
Histological brain tissue examination. Many dark blue granulocytes and macrophages (indicated by short, white arrows) diffusely infiltrated in the abscess area in (**A**), group A and (**B**), group B. After treatment with vancomycin-embedded poly(lactide-*co*-glycolide acid) microparticles, few inflammation cells were observed in (**C**), group C. Some residual particles were observed (indicated by black arrows).

At least one rat was euthanized, and brain tissues were obtained surgically 4 weeks after abscess aspiration and treatments in each group. Inflammatory response intensity was assessed using hematoxylin and eosin-stained section. Figure 9 presents the microscopic pathologic examination images for each group. In groups A (no treatment) and B (intraperitoneal administration with vancomycin), evident cerebral necrosis and inflammatory response with diffuse infiltration by mononuclear (MN) and polymorphonuclear (PMN) leukocytes were observed. In group C (treatment with stereotactic injection of VMPs), the MN and PMN leukocyte infiltration area was diminished. Some residual PLGA particles were observed (Figure 9c, marked with black arrows).

## 4. Discussion

Brain abscesses can occur in any location in the brain, and computed tomography (CT) and MRI are the major diagnostic methods to confirm their exact location [18,19]. Primary treatment should commence with broad spectrum antibiotics that cross the BBB in effective concentrations. Once antibiotic sensitivity tests and pus culture data are available, specific bactericidal agents for the cultured organism should be administered [2,4,20,21]. Image-guided stereotactic aspiration of the purulent center provides both diagnostic and therapeutic efficacy in treating brain abscesses [22,23]. Stereotactic aspiration can be performed under local anesthesia. This technique ensures that the injury to the brain surrounding the abscesses is as small as possible, resulting in minimal retrogression of the already-existing edema [23,24]. Stereotactic aspiration can be repeated safely and is considered the procedure of choice in cases of multiple and/or deeply-seated abscesses, for which an open surgical approach is impossible and/or perilous, and when the damage is situated in crucial areas of the brain [2,4,10]. Furthermore, advanced CT- or MRI-guided navigation systems can be used to create a three-dimensional real-time reconstruction intraoperatively. Meticulous trajectory planning can then be optimized from the point of brain entry to the abscess to avoid injury to areas of critical function [2,25]. Because of advances in image-guided stereotactic aspiration, an increasing number of institutes and neurosurgeons recommend early surgical intervention; its rapid recovery provides early improvement in clinical symptoms [24,26,27] and a considerable effect on brain abscess treatment has been demonstrated. Before the application of CT-guided stereotactic techniques, the mortality rate of brain abscess was 18%. The advent of CT-guided stereotactic surgery has enabled neurologists to reach near-zero mortality with little morbidity [24,25,28].

Vancomycin is a large glycopeptide compound with a molecular weight of approximately 1450 Da that can penetrate into most spaces of the body, including the BBB [29]. However, the concentrations acquired are variable and dependent on the body tissues and the degree of inflammation present. In studies examining the penetration of vancomycin into the cerebrospinal fluid (CSF) of uninflamed meninges, relatively low concentrations were obtained (range: 0–3.45 mg/L), representing CSF:serum ratios of 0–0.18. As expected, inflamed meninges improve penetration of vancomycin into CSF, with reported concentrations of 6.4–11.1 mg/L and CSF:serum ratios of 0.36–0.48 [10,30,31]. In our study, we stereotactically injected biodegradable PLGA VMPs into healthy rat brains with low doses of vancomycin (about 0.5 mg vancomycin). The in vivo concentration study revealed that vancomycin can be sustainably released from the VMPs and remains high for more than 8 weeks, with high brain:serum ratios ranging from 25.84 to 53.08.

Vancomycin exhibits time-dependent inhibition of susceptible bacteria. The drug concentration must be maintained above the minimum inhibitory concentration (MIC) for the majority of dosing intervals. Literature has recommended maintaining the peak value at 5–8 times the MIC and trough value 1–2 times the MIC [30,32]. Many literatures have suggested that the area beneath the concentration–time curve (AUC:MIC ratio) may be the pharmacodynamics index that best correlates with successful outcomes related to the administration of vancomycin [33]. An AUC:MIC value of ≥400 was associated with a successful outcome, whereas an AUC:MIC value of <400 correlated with a lower eradiation rate and higher mortality rate [33,34]. In addition, trough serum level monitoring is the most precise and functional method of monitoring vancomycin serum level. Raising trough concentration to 15–20 mg/L to reach the target AUC:MIC ratio is desirable but has not currently been supported by clinical trials [30]. Other possible treatments should be researched in patients with *S. aureus* infections that exhibit a vancomycin MIC of 2 mg/L or greater, due to the fact that achieving the desired AUC:MIC ratio (≥400) is not likely in this setting. However, increasing the dosage to obtain higher trough levels may raise the potential for toxicity [30,35]. In the present study, the AUC:MIC level was 1319.66 at day 3 and reached its maximum value (3410.96) at week 6, the AUC:MIC value of 2495.71 was maintained the end of the study (week 8). Local delivery systems can achieve therapeutic concentration in the CNS with extremely low agent usage.

By using a biodegradable PLGA as a vehicle for local delivery of vancomycin to the target site we can easily attain AUC:MIC ratios much greater than 400 and strengthen the therapeutic efficacy of antibiotics while preventing conceivable toxicity or potential side effects related to intravenous administration. Furthermore, the treatment period of intravenous antibiotic is shortened, and the resource use associated with intravenous antibiotic therapy, hospitalization, and repeated imaging examinations can be minimized. Delivery of antibiotics via biodegradable polymers is thus highly desirable for brain abscess treatment [36,37].

PLGA is highly biocompatible and biodegradable, exhibits a wide range of degrade times, has tunable mechanical properties, and most notably, is an FDA-approved polymer. PLGA is among the most attractive polymeric candidates for fabricating devices for drug delivery and tissue engineering applications in the past two decades [14,38,39]. Degradation of PLGA results in lactic and glycolic acids, which lastly degrade into CO_2_ and H_2_O [14,38]. The 50:50 PLGA-based drug delivery carrier can sustainably release high concentrations of therapeutic agents to the target area for more than 8 weeks and result in minimal inflammatory reactions in the brain [14,40]. The duration is longer than the recommended antibiotic treatment period (approximately 4–8 weeks) [3,4,40]. The injected amount of VMPs is low (2.5 mg of PLGA containing 0.5 mg of vancomycin) and does not cause mass effect after injection.

Electrospraying is a technique of producing micro-/nanoparticles by utilizing a high voltage electric field to break up a solution. Other techniques for the production of drug particles include spray drying, coarcevation, emulsion techniques, and solvent evaporation, and face challenges such as low drug-loading efficiency, particle size poly-dispersity, low ability to fabricate small particles, and difficulties for incorporation of hydrophilic drugs, electrospraying is becoming an attractive process for particle preparation as it surmounts various challenges confronting the technology. The drug loading efficiency of electrosprayed particles is theoretically 100%, yet the drug loading percentage of sprayed VMPs in this study may be less-than-optimal, since some particles may be ejected outside the collector and not totally collectable. Despite this, the VMPs can be easily produced by the process, after, they are which mixed with DMSO solution and introduced into the brain by using a stereotactic technique. Craniotomy or craniectomy is unnecessary, and therapeutic concentrations can be readily achieved in the brain by using a simple, less invasive method. Advanced CT- or MRI-guided navigation systems provide safer and more accurate alternative treatments for brain abscesses.

## 5. Conclusions

The results of this study demonstrated that VMPs can be introduced into the brain parenchyma using a stereotactic method and sustainably released high concentrations of vancomycin. The VMPs also achieved much higher AUC:MIC ratios than the recommended value (> 400) at the target site, whereas much lower drug concentrations were maintained in the blood. Serial brain MRI revealed that brain abscess volumes were significantly smaller in the VMP treatment group (group C) than those in the i.e., (group A) and intraperitoneal injection groups (group B). At end of the study (9 weeks posttreatment), 2, 5, and 12 rats survived in groups A, B, and C, respectively. The Kaplan–Meier analysis also revealed that the abscess-bearing rats in group C had a significantly longer survival time than those in groups A and B. These results suggest that electrosprayed VMPs are a favorable candidate for sustained release of antimicrobial agents to cure brain abscess.
